# Large‐Scale Study Comparing Analytical and Diagnostic Quality of Three HPV Self‐Sampling Devices for At‐Home Cervical Cancer Screening

**DOI:** 10.1111/apm.70109

**Published:** 2025-12-03

**Authors:** Alexander Arum, Emilie Korsgaard Andreasen, Helle Pedersen, Trine Dahl Nielsen, Peter Frandsen, Birgitte Tønnes Pedersen, Estrid Hogdall, Jesper Bonde

**Affiliations:** ^1^ Department of Pathology, afs. 134, AHH‐Hvidovre Hospital Copenhagen University Hospital Hvidovre Denmark; ^2^ Department of Pathology, HGH‐Herlev Gentofte Hospital Copenhagen University Hospital Herlev Denmark

## Abstract

Elimination of cervical cancer requires broad access to and participation in cervical screening. Self‐sampling has emerged as a robust technology that simplifies access; however, few self‐sampling devices are independently validated regarding sample quality. This study compares the quality of three self‐sampling devices: Evalyn (Rovers), currently used in Denmark, the FLOQSwab (Copan) and the modified FLOQSwab: SensiGrip. Women residing in the Capital Region of Denmark, accepting screening by self‐sampling, were offered a kit containing the following combinations of devices: (1) Evalyn, FLOQSwab, (2) Evalyn, SensiGrip or (3) FLOQSwab, SensiGrip. Returned kits were analyzed using the validated BD Onclarity HPV assay on the COR instrument (BD). A total of 1677 women participated. Sample quality was similar across devices, also when stratifying into age groups. Two hundred thirty‐two women (13.9%) tested positive for one or more oncogenic HPV types. Pairwise concordance analysis for each group showed an overall agreement between 93.5% and 95%. Analytical and diagnostic performance of samples collected by the three self‐sampling devices resulted in similar quality and HPV detection. Hence the sample quality is not a determinant in the choice of sampling device and focus on other factors such as cost, women's preference or size and weight can take precedence.

**Abbreviations**
Analytically: no cut‐off values are applied
CBD: cervical brush diluent‐buffer
CIN: cervical intraepithelial neoplasia
Diagnostically: Ct‐value cut‐off of ≤ 38.3 for HPV 16 and ≤ 34.2 for other HPV types is applied
Diluent tube: BD Onclarity HPV Self Collection Diluent Tube
HBB: human betaglobin
HPV: human papillomavirus
HR‐HPV: high risk human papillomavirus
SensiGrip: FLOQSwab SensiGrip

## 1 Introduction

Globally cervical cancer is the fourth most frequent cancer among women, but with large incidence and mortality disparities. Established screening and vaccine programs in high‐income countries have effectively reduced cervical cancer incidence whereas low‐income countries with limited preventive services continue to have a high incidence. Persistent infections with oncogenic human papillomavirus (HPV) are the primary cause of cervical cancer and the combined strategy of screening, treatment and vaccination against oncogenic HPV is central to the World Health Organization goal to eliminate cervical cancer [1, 2].

A successful cervical cancer elimination requires broad accessibility and participation in screening programs. In this context, self‐collected cervico‐vaginal samples have several merits [3]. HPV self‐sampling can mitigate access challenges and in countries with screening programs, it recruits women who otherwise actively or passively deselect participation, because of time constraints or reluctance towards the pelvic exam necessary to collect a cervical sample [4]. Finally, HPV self‐sampling in combination with quality assured and controlled HPV testing is a reliable alternative to the traditional cell‐based samples used in cervical screening [5, 6, 7, 8, 9, 10, 11, 12, 13].

Many devices and brush types are currently marketed globally for use in HPV self‐sampling. However, few self‐sampling devices have obtained diagnostic and analytical evidence at a level to support use in HPV self‐sampling programs. Among them, the Evalyn brush (Rovers, the Netherlands) is an HPV self‐sampling device, designed for comfort and ease of use and is employed in several programs. The Evalyn is designed with several safety features i.e., insertion indicator wings preventing too deep insertion and emitting an audible click with each turn of the brush guiding the woman during the sampling procedure. The brush is CE marked. Similarly, the FLOQSwab (Copan, Italy) is designed with a nylon fiber swab head, built to trap, and preserve the sample material. The FLOQSwab is CE certified, and variations are used in a wide range of healthcare applications. Compared to the Evalyn brush, the FLOQSwab is smaller and more compact consisting of a simple dry swab and transport container. Globally, the Evalyn brush and FLOQSwab are the most widely used self‐sampling devices for HPV self‐sampling. Finally, a new HPV self‐sampling version of the FLOQSwab has been developed, the FLOQSwab SensiGrip (SensiGrip; Copan, Italy). The SensiGrip is a modified FLOQSwab, designed to make cervical sampling easier, simpler, and safer.

Substantial quality assurance and control has been documented for HPV testing on self‐collected samples [14, 15], and the diagnostic parity of HPV self‐samples compared to clinic‐collected samples is well‐established when used in combination with equally quality‐assured and controlled HPV tests [16]. Additionally, the analytical stability of self‐samples as a function of time and temperature has shown that even extended time between sampling and testing that is, transport time to the laboratory, is acceptable given that the HPV self‐sample is shipped in a dry format without resuspension in the hands of the user [17]. However, a research gap exists detailing the sample quality derived from different self‐sampling brushes and swabs in real‐life settings.

Here we conducted a large‐scale study evaluating the analytical and diagnostic quality of three HPV self‐sampling devices: the Evalyn, the FLOQSwab and the SensiGrip, as potential alternative self‐sampling devices in screening programs.

## 2 Materials and Method

### 2.1 Study Setting

Cervical cancer screening is offered to all resident women 23–64 years old by the Danish healthcare system and free of charge for women. HPV self‐sampling is an integrated element in the cervical screening program. Subgroups of women offered HPV self‐sampling include:

- Screening non‐attenders without screening attendance for more than one age defined interval.
- As a free‐of‐choice to women with the 2nd screening reminder 6 months after the initial invitation.
- As a free‐of‐choice to women offered HPV self‐sampling an alternative to a clinic collected sample with first screening invitation (study setting).

Women eligible for HPV self‐sample in either routine screening or study setting received an invitation by digital mail. To obtain an HPV self‐sampling kit, women were asked to register online using a dedicated web‐platform and unique code included in the digital mail. If registering for an HPV self‐sample, women were offered the opportunity to participate in this quality development study.

### 2.2 Study Design

This prospective diagnostic accuracy study was nested into the screening program of the Capital Region of Denmark. Participating women received an HPV self‐sampling kit with two sampling devices, information material, instructions, and a prepaid return envelope. Devices were distributed in three combinations: (1) Evalyn and FLOQSwab, (2) Evalyn and SensiGrip swab or (3) FLOQSwab and SensiGrip swab. Women were asked to provide the date of sampling and were given written instructions regarding the self‐sampling procedure, allowing for an analysis of biostability over time.

Sampling order was randomized and allocation to sample groups was done in consecutive batches as women registered their participation in the study. In group 1 (FLOQSwab), and group 2 (SensiGrip), a BD Onclarity HPV Self Collection Diluent Tubes (forthwith “diluent tube”) was provided for the women to transfer the swab to the diluent tube immediately after sampling. In group 3, since the two nearly identical swabs could easily be mixed, the FLOQSwab and Sensigrip were returned in the standard container and processed in the laboratory upon reception. The two modes of return to laboratory of FLOQswab and SensiGrip in sheath or diluent tube allows for analysis of the biostability in both containers during transport. All Evalyn brushes were returned with cap on and in an included sealed plastic bag. Samples were returned via the postal service.

The study is reported as per STARD guideline.

### 2.3 Sample Processing

At the Department of Pathology, AHH‐Hvidovre Hospital, all received Evalyn brush heads were manually transferred to a BD tube containing 3.0 mL of cervical Brush Diluent‐buffer according to standard operating procedure. FLOQSwab and SensiGrip in group 3 were transferred to a diluent tube without buffer, identical to the one handled by the sampling women in groups 1 and 2.

All analysis was done using the BD COR platform [14]. 3.0 mL of Cervical Brush Diluent‐buffer (CBD) was automatically added to FLOQSwabs and Sensigrips and all samples were subsequently analyzed in accordance with the manufacturer's specifications for the BD Onclarity HPV assay [14, 18, 19, 20]. The BD Onclarity HPV assay is designed with a Ct‐value cut‐off, of ≤ 38.3 for HPV16 and ≤ 34.2 for other HPV types. When applying the cut‐off, results are described as “diagnostic”, when not applying the cut‐off value, results are described as “analytical” [21].

All test results including Ct‐scores for the internal control human beta‐globin (HBB) and HPV were retrieved from the BD COR. Invalid results were reanalyzed once. If the sample remained invalid, the output was registered as either INC: inconclusive, ICF: Internal control failure or LLF: Liquid level failure. Invalid samples were excluded from analyses.

### 2.4 Statistical Analysis

Because Evalyn has been locally validated and is currently used in the Danish cervical cancer screening program, and Evalyn was used as the reference standard for groups 1 and 2 and FLOQSwab and SensiGrip as index tests and FLOQSwab as the reference for group 3 and SensiGrip as the index test.

Internal control (humane beta globin) Ct‐values were interpreted as a surrogate marker for cellularity of the samples and were pooled across assay wells and study groups. Subsequently, the Ct‐values of each group were stratified according to days between sample and analysis, and into age groups of the sampled women. Results were conveyed in boxplots, tables, and Bland–Altmann plots.

Invalid samples were excluded and pairwise comparison of HR‐HPV detection (positive or negative) was performed. In the analytical comparison, any Ct value was interpreted as positive. Pairwise reliability was calculated as kappa values, overall agreement, percent positive agreement and percent negative agreement. Additionally, a pairwise noninferiority analysis of sensitivity with a delta value of 0.95 was calculated following established guidelines [22].

Diagnostic and analytical outcomes were illustrated in contingency tables. HPV positive tests were stratified into HPV types, and overall diagnostic and analytical agreement as well as kappa values were calculated.

Data wrangling and graphs were created using Python (version 3.10.5) using the pandas, matplotlib, seaborn packages. Statistical analysis was done in R (version 4.4.1); a non‐inferiority test was done in Excel.

## 3 Results

**FIGURE 1:**
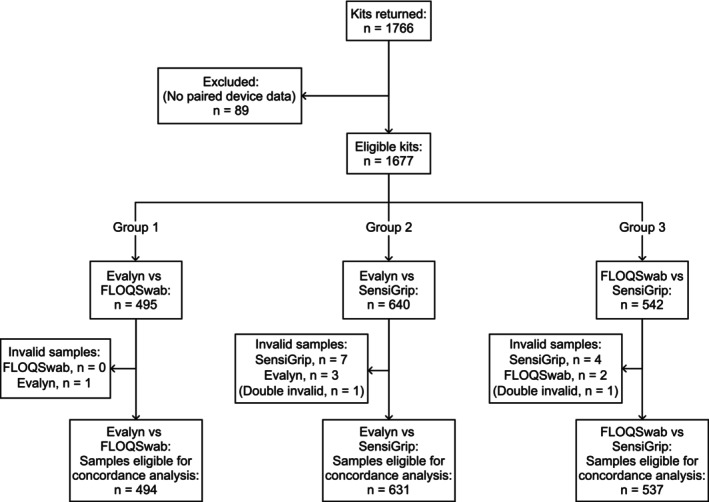
Modified STARD flowchart, number of returned kits, and number of samples eligible for concordance analysis.

A total of 1766 kits, each containing two paired devices, were returned for analysis. Out of these, 1677 contained adequate data after analysis by daily staff, for it to be retrieved and merged into the study dataset (Figure 1, Group 1: Evalyn and FLOQSwab, *n* = 495; Group 2: Evalyn and SensiGrip *n* = 640; and Group 3: SensiGrip and FLOQSwab, *n* = 542, respectively). When pooled across groups, a total of 1135 Evalyn brushes (5 invalid results), 1037 FLOQSwab (4 partially invalid results) and 1182 SensiGrip swabs (11 invalid results) were included in the analysis.

**FIGURE 2:**
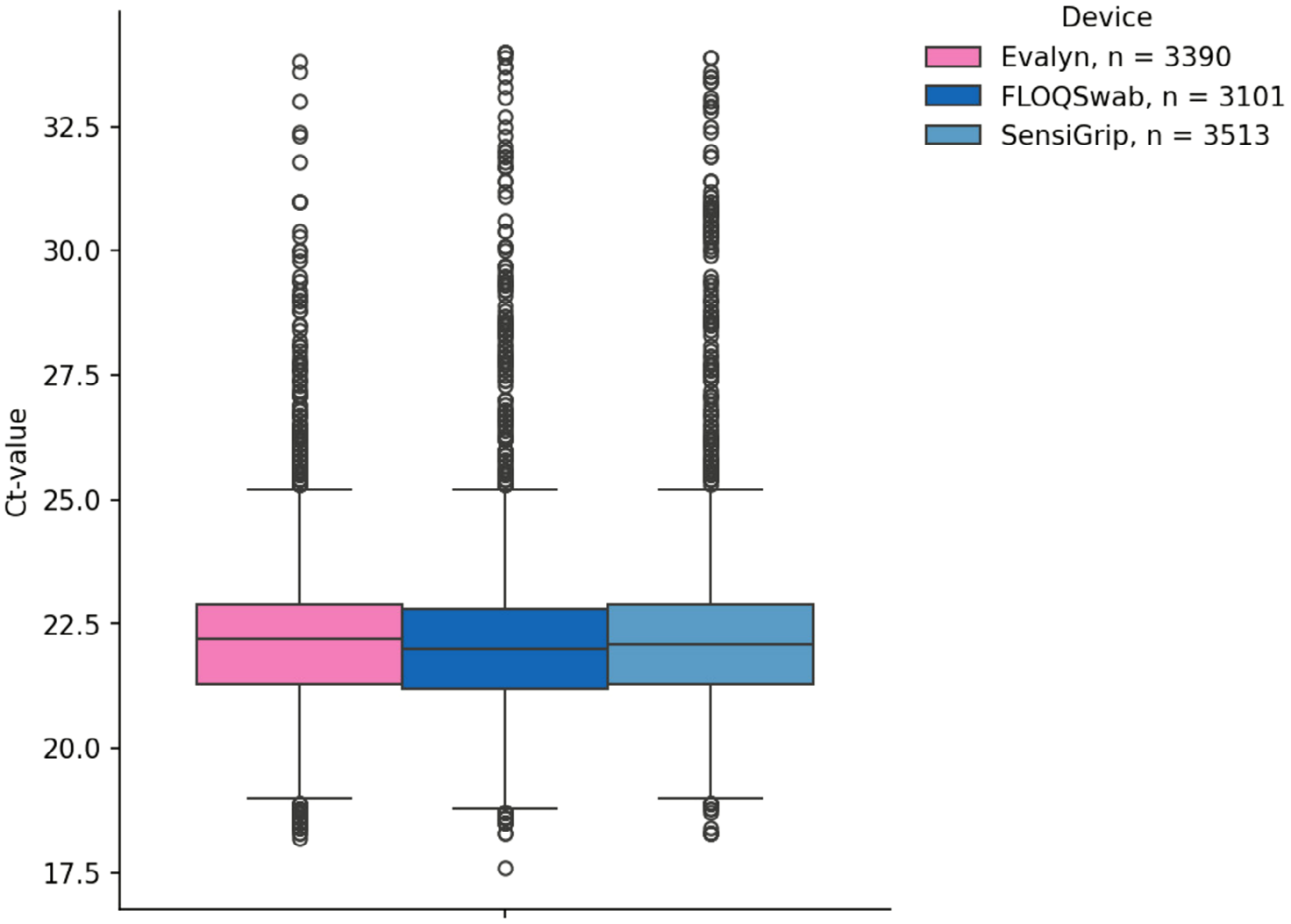
Internal control Ct‐values of each device, pooled across groups.

The Onclarity HPV assay is designed to output 3 internal control Ct‐values per sample. Pooling devices across groups, resulted in a total of 3390 datapoints from Evalyn brushes, 3101 datapoints from FLOQSwab and 3513 datapoints from SensiGrip. Evalyn had a median HBB Ct‐value of 22.2 with a mean of 22.2 (SD: 1.6), FLOQSwab had a median HBB Ct‐value of 22.0 and a mean of 22.2 (SD: 1.9), while SensiGrip had a median HBB Ct‐value of 22.1 and a mean of 22.3 (SD: 1.9) (Table 1, Figure 2).

**TABLE 1:** Descriptive statistics regarding Ct‐values of internal controls of individual brushes and stratified by age groups.

			Count	Mean	Std	Median
Evalyn	Age group	21–30	468	22.0	1.64	21.8
		30–39	855	22.2	1.53	22.2
		40–49	792	22.5	1.73	22.4
		50–59	873	22.1	1.65	22.2
		> 60	402	22.0	1.53	22.1
		Total	3390	22.2	1.63	22.2
FLOQSwab	Age group	21–30	546	22.0	1.63	21.9
		30–39	801	22.3	1.81	22.1
		40–49	626	22.5	1.69	22.4
		50–59	732	22.4	2.19	22
		> 60	396	21.8	2.09	21.5
		Total	3101	22.2	1.91	22
SensiGrip	Age group	21–30	555	22.1	1.70	21.9
		30–39	882	22.3	1.70	22.2
		40–49	756	22.7	2.16	22.4
		50–59	840	22.4	1.92	22.1
		> 60	480	22.0	1.68	21.8
		Total	3513	22.3	1.87	22.1

**FIGURE 3:**
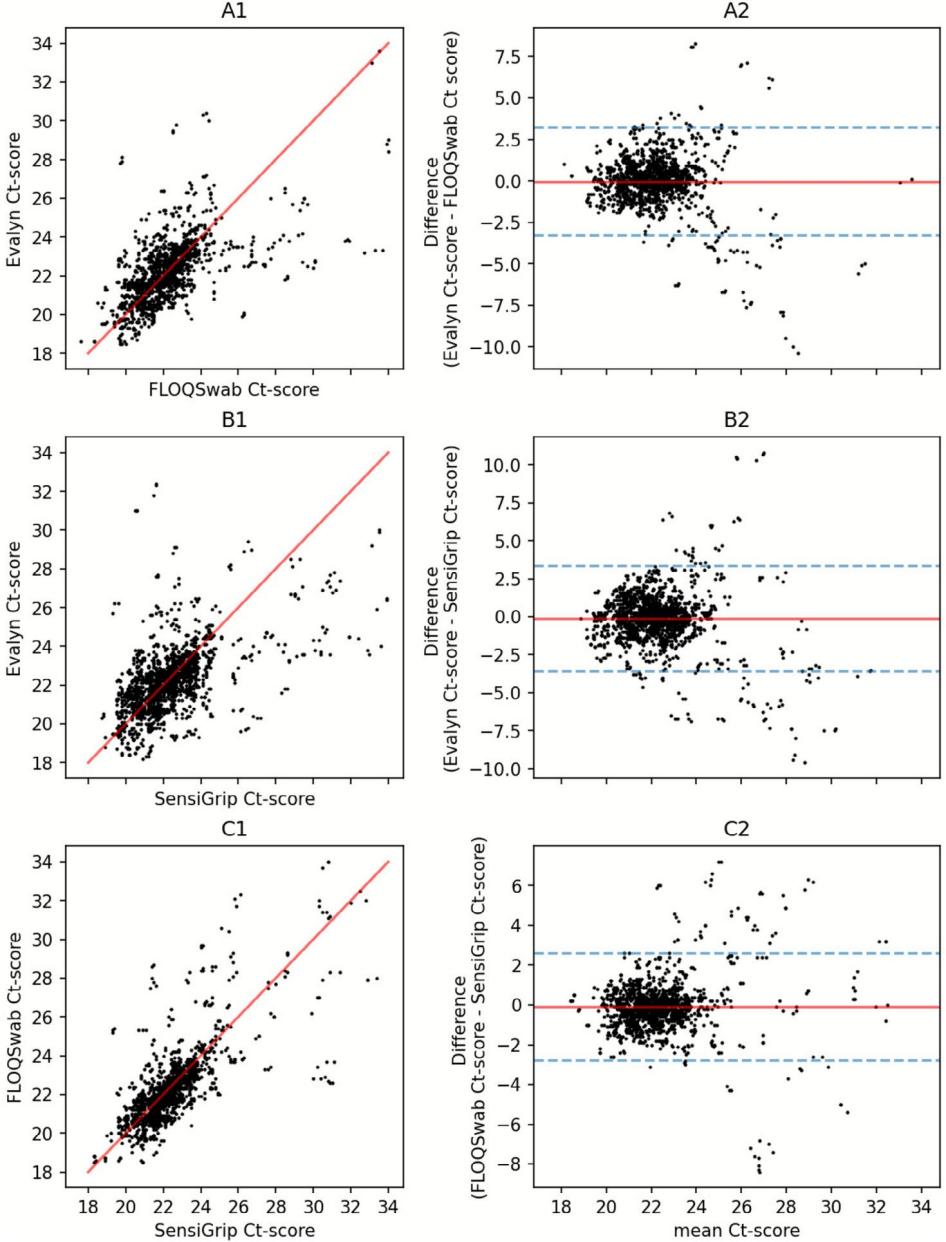
Ct‐scores and Bland–Altman plots of Ct‐scores of each group. (A1) Internal control Ct‐values, Evalyn (Reference) *n* on *y*‐axis and FLOQSwab (Index) on *x*‐axis. (A2) Bland–Altman plot, Evalyn—FLOQSwab. Mean difference: −0.02, upper limits of agreement: 3.22, lower limits of agreement: −3.27. (B1) Internal control Ct‐values. Evalyn (Reference) on *y*‐axis and SensiGrip (Index) on *x*‐axis. (B2) Bland–Altman plot, Evalyn—SensiGrip. Mean difference: −0.12, upper limits of agreement: 3.33, lower limits of agreement: −3.57. (C1) Internal control Ct‐values. FLOQSwab (Reference) on *y*‐axis and SensiGrip (Index) on *x*‐axis. (C2) Bland–Altman plot, FLOQSwab–SensiGrip. Mean difference: −0.09, upper limits of agreement: 2.61, lower limits of agreement: −2.79.

When comparing paired internal control Ct‐values a near‐linear relationship was observed, and when plotted into Bland–Altman plots, mean differences of −0.12 or less were observed with narrow limits of agreement (Figure 3).

**FIGURE 4:**
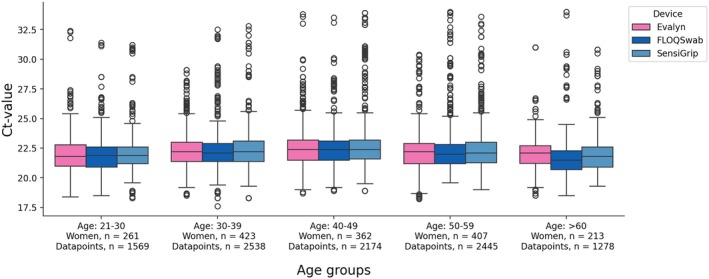
Internal control Ct‐values of each device, pooled across groups, and stratified into age groups.

When stratifying internal control Ct‐values into age groups of the sampling women, there was a statistically significant difference of 0.6 Ct‐values that falls within acceptable variation (Figure 4).

Sampling order did not show any differences regarding internal control Ct‐values between the 1st and the 2nd collected device (data not shown). Also, for FLOQSwab and SensiGrip, whether shipping in either FLOQSwab standard tube (sheath) or BD test tube did not influence Ct‐values (data not shown).

**FIGURE 5:**
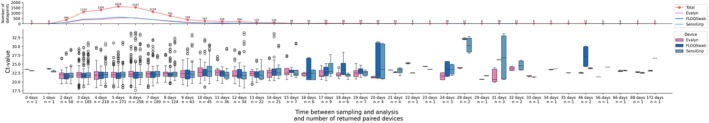
Internal control Ct‐values of each device, pooled across groups and stratified into time between sampling and analysis.

Analytical stability over time of the three devices was assessed using the self‐reported sampling date and the date of analysis. We observe that 95% of samples were returned within 14 days after the self‐reported sampling date. Using the HBB Ct score, we see that the Ct‐values remain stable across devices for this period; intervals beyond two weeks contained too few datapoints to be conclusive (Figure 5).

In total, 1662 samples completed analysis for HPV and diagnostically 232 (13.9%) tested positive for at least one HR HPV‐type on at least one of two paired self‐sampling devices. When disregarding cut‐offs, 368 women (22.1%) tested positive (analytical PCR positive).

A pairwise analysis of analytical HPV agreement (without cut‐off) shows that Evalyn and FLOQSwab have an overall agreement of 93.5% (*n* = 494), Evalyn and the SensiGrip 94.1% (*n* = 631) and FLOQSwab versus SensiGrip was 95.0% (*n* = 537), respectively (Table 3). Evalyn and FLOQSwab had a positive agreement of 84.7%; Evalyn and the SensiGrip 92.4%; FLOQSwab versus SensiGrip 87.3%. Similarly, negative agreement was found as 95.4%, 94.5%, and 96.8%, respectively. We found that Evalyn vs. FLOQSwab and Evalyn vs. SensiGrip were analytically non‐inferior, whereas SensiGrip and FLOQSwab were evaluated as inferior (Table 3).

Diagnostically, Evalyn and FLOQSwab had an overall agreement of 97.8% (*n* = 494), Evalyn and the SensiGrip 96.5% (*n* = 631) and FLOQSwab versus SensiGrip was 97.4% (*n* = 537), respectively (Table 3). Evalyn and FLOQSwab had a positive agreement of 89.4%, Evalyn and SensiGrip 89.4%, FLOQSwab versus SensiGrip 85.3%. Negative agreement was 98.9%, 97.6% and 99.1%, respectively. Diagnostically Evalyn vs. SensiGrip was evaluated as non‐inferior and Evalyn vs. FLOQSwab and FLOQSwab vs. SensiGrip, as inferior (Table 4).

When stratifying diagnostic results into individual HPV types and HPV groups, overall diagnostic and analytical agreement was 98% or above for all three combinations of devices, and kappa values were evaluated as either good or excellent, with the majority being excellent (Table 2).

**TABLE 2:** Combined diagnostic outcomes, analytical agreement, diagnostic agreement and diagnostic kappa value for each combination of devices and stratified into HPV types.

HPV type	Diagnostic				Analytical agreement (%)	Diagnostic agreement (%)	Diagnostic kappa value
	Evalyn/FLOQSwab						
	−/−	+/+	+/−	−/+			
HPV 16	485	7	1	1	99.2	99.6	0.87
HPV 18	494	0	0	0	99.2	100	1
HPV 31	491	3	0	0	99.4	100	1
HPV 45	487	6	1	0	98.8	99.8	0.92
HPV 51	484	8	1	1	98.4	99.6	0.89
HPV 52	488	3	2	1	99.6	99.4	0.66
HPV 33/58	484	9	1	0	99.6	99.8	0.95
HPV 56/59/66	474	12	5	3	97.6	98.4	0.74
HPV 35/39/68	478	13	1	2	99	99.4	0.89
	Evalyn/SensiGrip						
HPV 16	615	11	4	1	99.4	99.2	0.81
HPV 18	627	2	2	0	99.7	99.7	0.67
HPV 31	628	3	0	0	99.5	100	1
HPV 45	624	6	1	0	99.7	99.8	0.92
HPV 51	619	9	0	3	98.4	99.5	0.85
HPV 52	620	9	2	0	99.5	99.7	0.9
HPV 33/58	621	9	1	0	99.8	99.8	0.95
HPV 56/59/66	598	22	3	8	98.1	98.3	0.79
HPV 35/39/68	605	19	3	4	97.3	98.9	0.84
	FLOQSwab/SensiGrip						
HPV 16	527	7	1	2	99.1	99.4	0.82
HPV 18	536	1	0	0	99.4	100	1
HPV 31	532	3	1	1	98.9	99.6	0.75
HPV 45	531	4	2	0	99.4	99.6	0.80
HPV 51	524	11	2	0	98.7	99.6	0.91
HPV 52	525	11	0	1	99.4	99.8	0.96
HPV 33/58	528	6	1	2	99.4	99.4	0.80
HPV 56/59/66	519	13	3	2	98.7	99.1	0.83
HPV 35/39/68	522	12	2	1	98	99.4	0.89

*Note:* Diagnostic results are analyzed according to BD manufacturer set threshold (Ct values ≤ 38.3 for HPV16 and ≤ 34.2 for every other type of HR‐HPV counted as positive; samples above Ct cut‐off are counted as negative) Analytical agreement is evaluated without a threshold value. Kappa values were categorized as follows: 0.00–0.20: Poor; 0.21–0.40: Fair; 0.41–0.60: Moderate; 0.61–0.80: Good; 0.81–1.00: Excellent [23].

## 4 Discussion

HPV self‐sampling has proven to mitigate several barriers to cervical cancer screening participation, including, but not limited, to bypassing the availability of primary care facilities and subsequent pelvic examination, reduced cost compared to clinician‐collected samples, and simplified logistics since they can be distributed via mail, pharmacies or community health centers for the purpose of improving women's access to cervical cancer screening. However, for HPV self‐sampling to live up to its promise, the quality of HPV testing must be on par with clinician‐collected samples. This parity is best achieved when assessing the workflow in its entirety, sample quality, suspension method, and the HPV analysis.

In Denmark, the Evalyn brush is the current standard of choice for self‐sampling in the cervical cancer screening program. Any change in self‐sampling device must rely on evidence of quality improvement and/or women's preferences in device design. The Evalyn brush design has been validated in combination with the BD‐Onclarity HPV assay [23], and studies have documented the biological and analytical stability of samples collected using an Evalyn brush over time and at different temperatures [17]. In this study, we compared three self‐sampling devices with respect to analytical and diagnostic sample quality when (1) transported dry from woman to laboratory [17, 24], (2) when suspended into 3 mL CBD media [10], (3) when analyzed using the BD Onclarity HPV test [19, 20, 24], and (4) when analyzed on the high throughput BD COR test platform [14].

Sampling order did not influence sample quality. Also, no effect of the woman's age regarding sampling quality was observed (as per HBB Ct scores). The latter is an improvement over cytology, where age associated cervical atrophy can complicate microscopic evaluation [25].

Medians, and interquartile ranges of the three self‐sampling devices were similar regarding HBB Ct‐values (Table 1, Figure 2) and regarding HPV detection (Table 2). Here, we conducted the analysis analytically for HPV positivity and diagnostically with the cut‐off applied. In both cases, the pairwise agreements were 94% or better, indicating that the resulting sample quality using this flow is similar between devices (Table 3). The distinction between analytical and diagnostic may seem irrelevant, as it is in this case. However, it can be envisioned that similar quality assessments may be impacted by the manufacturer‐set cut‐offs for HPV positivity given that the cut‐offs are typically set against a proficiency test or in the case of international clinical validation, against detection of cervical intraepithelial neoplasia grade 2 (CIN) or worse in clinic‐collected samples [22, 26, 27, 28, 29]. It is therefore not unexpected to observe differences in analytical or diagnostic sample quality if a clinical HPV self‐sampling test work flow is applied without attention to the combination of device, diluent/dilution factor, HPV test and test platform [5, 30].

**TABLE 3:** Analytical concordance between the Evalyn brush paired with FLOQSwab, Evalyn brush paired with SensiGrip generation swab device, and FLOQSwab paired with SensiGrip generation.

	Evalyn brush			
FLOQSwab		Positive	Negative	∑
	Positive	72 (14.57%)	19 (3.85%)	91 (18.42%)
	Negative	13 (2.63%)	390 (78.95%)	403 (81.58%)
	∑	85 (17.21%)	409 (82.79%)	494 (100.0%)
	Overall agreement			93.5%
	Non‐inferiority analysis			*p* = 0.03
	Evalyn brush			
SensiGrip		Positive	Negative	∑
	Positive	110 (17.43%)	28 (4.44%)	138 (21.87%)
	Negative	9 (1.43%)	484 (76.7%)	493 (78.13%)
	∑	119 (18.86%)	512 (81.14%)	631 (100.0%)
	Overall agreement			94.1%
	Non‐inferiority analysis			*p* = 2.86e−05
	FLOQSwab			
SensiGrip		Positive	Negative	∑
	Positive	90 (16.76%)	14 (2.61%)	104 (19.37%)
	Negative	13 (2.42%)	420 (78.21%)	433 (80.63%)
	∑	103 (19.18%)	434 (80.82%)	537 (100.0%)
	Overall agreement			95.0%
	Non‐inferiority analysis			*p* = 0.12

*Note:* All tests with an HPV Ct‐value ≤ 40 cycles are counted as positive (regardless of genotype detected). Only samples without Ct‐values ≤ 40 are counted as negative.

In terms of biostability, we found samples to be analytically stable up to two weeks between sample and analysis (Figure 5), however with the caveat that our analysis is limited by the number of data points extending further than two weeks. Also, no difference in biostability was observed when SensiGrip or FLOQSwab was returned to lab analysis in a diluent tube instead of the standard sheath. A previous in situ study has shown longer biostability of a dry Evalyn self‐sample for HPV testing [17]. Also, a clinical study showed segregated results based on the time between collection and specimen processing in the laboratory. Here, if the time between specimen collection and preparation was 28 days or less, the Copan swab had similar sensitivity for the detection of CIN 3 + as a clinic‐collected sample [16]. In our setting, we hypothesize that participating women were more conscious regarding returning the self‐sample kits given the nature of participation in a clinical trial, and hence the return after sampling was swifter than can be expected in an everyday clinical setting. Given the limitation to assess biostability in this set up, we caution that biostability can be substantially longer for dry transported samples using any of the three devices evaluated in our study.

Current literature describes the ideal reference standard, which in the case of new HPV assays for HPV‐based screening entails international validation [22, 26, 31]. For HPV self‐collected samples, the validation framework is less extensive with only one international consensus protocol [32]. For cervical cancer screening, the current reference standard for new HPV assays is to be assessed against known histological diagnosis of CIN2/CIN3. In this study, the use of CIN2/CIN3 and the necessary paired sampling by a clinician, was considered inexpedient. Though a recent systematic review shows that most studies are done with clinician‐collected samples as reference, a few have adopted validated self‐sample devices as legitimate references [14, 33]. HR‐HPV sampling, including self‐samples, has proven equal to clinician‐collected samples regarding sensitivity and specificity, when using validated PCR techniques and when applying manufacturer‐specified cut‐off values [6, 34]. Consequently, a validated self‐sampling device, analyzed with a validated assay can be considered a valid reference, a trend that we believe will increase, as confidence in self‐sampling technology increases.

When assessing the number of invalid results, we found slightly more invalid test results with the SensiGrip. As the SensiGrip swab head is identical to the FloqSwab, we find it unlikely that this is an effect of manufacturing. Instead, it could be either a stochasticity or relating to women's way of sampling with the two products. However, in the grand scale of things, the invalidity level is not a cause for concern.

Diagnostic and analytical output shows high levels of agreement between all combinations of the three devices. However, when testing non‐inferiority, Sensigrip is found inferior to FLOQSwab both when all samples with a Ct score of ≤ 40 as thresholds are counted (analytical, Table 3) and when the manufacturer set HPV cut‐off (clinical, Table 4) is applied. The inferiority calculations are in both cases driven by proportionally few cases of negative/positive and positive/negative (Tables 3 and 4, Sensigrip versus FLOQSwab) and can be interpreted as a weakness of the non‐inferiority analysis which is widely used within the field of cervical cancer prevention and screening [22, 35]. Nevertheless, for all practical purposes we consider the outcome to show that the brushes perform similarly if not identically.

**TABLE 4:** Diagnostic concordance between the Evalyn brush paired with FLOQSwab, Evalyn brush paired with SensiGrip, and FLOQSwab paired with SensiGrip generation.

	Evalyn brush			
FLOQSwab		Positive	Negative	∑
	Positive	51 (10.32%)	5 (1.01%)	56 (11.34%)
	Negative	6 (1.21%)	432 (87.45%)	438 (88.66%)
	∑	57 (11.54%)	437 (88.46%)	494 (100.0%)
	Overall agreement			97.8%
	Non‐inferiority analysis			*p* = 0.29
	Evalyn brush			
SensiGrip		Positive	Negative	∑
	Positive	76 (12.04%)	13 (2.06%)	89 (14.1%)
	Negative	9 (1.43%)	533 (84.47%)	542 (85.9%)
	∑	85 (13.47%)	546 (86.53%)	631 (100.0%)
	Overall agreement			96.5%
	Non‐inferiority analysis			*p* = 0.04
	FLOQSwab			
SensiGrip		Positive	Negative	∑
	Positive	58 (10.8%)	4 (0.74%)	62 (11.55%)
	Negative	10 (1.86%)	465 (86.59%)	475 (88.45%)
	∑	68 (12.66%)	469 (87.34%)	537 (100.0%)
	Overall agreement			97.4%
	Non‐inferiority analysis			*p* = 0.77

*Note:* All tests with HPV Ct registered according to BD manufacturer set threshold are counted as positive (≤ 38.3 for HPV16 and ≤ 34.2 for every other type of HR‐HPV). Samples above Ct cut‐off are counted as negative.

In conclusion, the three devices, Evalyn, FLOQSwab and SensiGrip, perform analytically and diagnostically similarly with respect to sampling quality and test results assessed by HPV detection. Also, no differences in biostability were observed in more than 1600 collected, paired samples when assessed by internal control Ct‐scores. With similar test performance between devices, decision makers' choice of device can instead be made based on factors such as cost, women's preference, and logistical parameters relating to mail‐based distribution such as size and weight of devices.

## Funding

This work was supported by Intramural funds.

## Ethics Statement

The Preference study is registered at the Regional Medical Ethical Committee for the Capital Region of Denmark, J. no. H‐2205‐6235, and by AHH‐Hvidovre Hospital, J. no. L‐21810‐80002. Electronic informed consent was obtained prior to inclusion in the study.

## Data Availability

Study data is archived at AHH‐Hvidovre Hospital; aggregated data can be made available by contacting the corresponding author.
